# Intensive Cryotherapy in the Emergency Department (ICED): A Randomized Controlled Trial

**DOI:** 10.5811/westjem.2020.10.48831

**Published:** 2021-01-21

**Authors:** Eric J. Leroux, Elizabeth A. Kaufman, Christian N. Kontaxis, Grant S. Lipman

**Affiliations:** * Eisenhower Health, Department of Emergency Medicine, Rancho Mirage, California; † Scripps Health, Department of Emergency Medicine, San Diego, California; ‡ Stanford University, Department of Undergraduate Studies, Stanford, California; § Stanford University School of Medicine, Department of Emergency Medicine, Stanford, California

## Abstract

**Introduction:**

Pain control is an essential component of musculoskeletal injury treatment in the emergency department (ED). We evaluated the most effective type of cryotherapy for analgesia of acute musculoskeletal injury and the impact on opioid utilization.

**Methods:**

This was a prospective, randomized, single-blind controlled trial of adult ED patients who presented with acute musculoskeletal pain. Patients were randomized to either intensive targeted cryotherapy (crushed wetted ice in a plastic bag) or agitated chemical cold pack applied to the injury site for 20 minutes. All other diagnostic and therapeutic orders were at the discretion of the treating physician. Visual analog pain scores were measured at the time of cryotherapy application, at 20 minutes (time of cryotherapy removal), and at 60 minutes (40 minutes after removal).

**Results:**

We enrolled 38 patients, 17 randomized to intensive targeted cryotherapy and 21 to chemical cold packs, with well-matched demographics. The intensive targeted cryotherapy group achieved significantly greater pain relief at 20 minutes (2.1 [95% confidence interval (CI), 1.3 – 2.9] vs 0.9 [95% CI, 0.3 – 1.5], P < 0.05) and at 60 minutes (2.7 [95% CI, 1.6 – 3.7] vs 1.2 [95% CI, 0.6 – 1.7], P < 0.05), number need to trial (NNT) = 3.2. Opioid administration in the ED was significantly lower in the intensive targeted cryotherapy group (1 [6%] vs 7 [33%], P < 0.05), NNT = 3.6. Those who received a discharge opiate prescription had significantly higher 60-minute pain scores (7.3 ± 2.2 vs 4.1 ± 2.7, P < 0.05).

**Conclusion:**

Intensive targeted cryotherapy provided more effective analgesia than chemical cold packs for acute musculoskeletal injuries in the ED and may contribute to lower opioid usage.

## INTRODUCTION

### Background

Musculoskeletal injuries are the most common class of presenting complaints for emergency department (ED) visits, with pain relief being the primary reason for seeking medical care.^1^ Cryotherapy is a non-pharmacologic therapy with analgesic properties first recognized by Hippocrates, and commonly used for acute musculoskeletal treatment and rehabilitation in athletes.^2^ The local analgesic effects of cryotherapy result from a decrease in nerve conduction velocities, edema formation, cellular metabolism, and local blood flow.^3^ Although there is no ideal post-cooling tissue temperature, consensus supports that greater and faster cooling improves pain relief.^3^

A common ED practice is the application of chemical cold packs (CCP) to the skin of the injured area for rapid analgesia. However, wetted crushed ice (intensive targeted cryotherapy, ITC) in a plastic bag has been found to produce lower skin surface temperatures than a CCP after 15–20 minutes.^3^–^5^ While ITC has been found to reduce inflammation and pain from acute musculoskeletal injuries, evidence supporting its role in the ED is scarce.^2^ During the current pandemic an estimated 1.7 billion people, or approximately one in five worldwide, have sheltered in place,^7^ with a reported weekly exercise increase of 88%.^8^,^9^ With increased exercise, it is reasonable to expect musculoskeletal pain; efficacious home analgesia could potentially prevent non-emergent hospital visits.

Opioid pain medications are often used for analgesia in the ED, with 15% of all adult patients from 2016–2017 receiving a prescription for opioids upon discharge.^10^ Of 35,000 ED patients seen for acute ankle sprains from 2011–2015 in the United States, 25% were prescribed opioids.^11^ It has been found that patients prescribed opioids were more likely to progress to prolonged use than those who were not.^12^ Intensive targeted cryotherapy in postoperative musculoskeletal patients resulted in fewer prescription analgesics,^13^ but this has not been studied in ED patients. The primary goal of this trial was to investigate whether ITC could provide more effective analgesia for acute musculoskeletal injuries than CCP, with a secondary goal of assessing the impact of cryotherapy on opioid usage.

## METHODS

### Study Design and Setting

This was a prospective, randomized, single-blind controlled trial conducted in an academic tertiary Level I ED with more than 70,000 annual patients. From February–April 2016, research assistants enrolled patients daily between 12 pm and 9 pm. The study was approved by the institutional review board (ClinicalTrials.gov: NCT02720315).

### Selection of Participants

Eligible patients were aged 18–65 presenting with complaints of acute musculoskeletal pain. They were identified based on chief complaint and triage note in the electronic health record. Exclusion criteria included trauma activation, patients with a known pregnancy, open fracture or obvious deformity likely to require closed reduction, hip fracture, altered mental status, or if the patient was receiving an investigational drug as part of an ongoing trial. Trained research assistants approached eligible participants in the waiting room where informed consent was obtained and cryotherapy initiated. Participants enrolled on even days of the month were randomized to CCP, and those on odd days received ITC.

### Interventions

Participants received either an activated CCP (MediChoice product #1480069904, Owens & Minor, Inc, Richmond, VA) applied to the skin at the site of injury, or wetted crushed ice that was double-bagged in thin, sealed plastic bags and wrapped in place by plastic wrap, both by an ED technician. Based on the thermodynamics of the CCP, optimal sustained cooling by CCP alone would require replacing the pack every nine minutes.^14^ However, in accordance with existing departmental protocol, the technician removed the respective cryotherapy modality after 20 minutes.

**Population Health Research Capsule**

What do we already know about this issue?

Anecdotes and literature from other fields of medicine demonstrate that cryotherapy has analgesic effects for patients with musculoskeletal (MSK) injuries.

What was the research question?

Does intensive targeted cryotherapy (ITC) relieve pain or reduce the need for opioid pain relievers in the ED?

What was the major finding of the study?

Compared to usual care, ITC is an effective analgesic and is associated with lower opioid utilization.

How does this improve population health?

Cryotherapy holds promise as a safe and effective alternative to opioids for patients with acute MSK injuries, and thus could help address the opioid epidemic.

### Methods of Measurement

Pain severity was measured using a validated 100-millimeter (mm) visual analog scale.^15^ Pain scores were obtained at three time points during the participant’s stay in the ED: immediately prior to applying the ice (0 minutes); immediately after cryotherapy removal (20 minutes); and 40 minutes after cryotherapy removal (60 minutes from the initial measurement). If patients were discharged before 60 minutes, a pain score was obtained upon discharge. The participants’ ED length of stay, discharge diagnoses, results of radiology studies, timing, and doses of medications received, patient disposition, and discharge medications were obtained via chart review from an author blinded to cryotherapy allocation.

### Outcome Measures

The primary outcome was the change in pain severity at 60 minutes, at which time tissue temperatures post-cryotherapy are shown to have returned to normal.^3^ Each patient was categorically classified as having obtained significant pain relief or not, at each time point. The minimum clinically significant change in patient pain was defined as 13 mm, regardless of initial pain severity.^16^ Secondary outcomes included the pain change at 20 minutes, administration of opioids or benzodiazepines in the ED, length of stay, and presence of discharge prescriptions containing opioids or benzodiazepines.

### Data Analysis

We calculated outcomes per intent-to-treat analysis. To achieve 80% power (μ = 0.05, 2-tailed test), 38 participants were required to detect a difference of 13 mm in pain severity score. Pain score change was analyzed by t-test, administration of opioids or benzodiazepines in the ED by Fisher’s exact test, and discharge prescriptions by χ^2^. P-values < 0.05 were considered significant and 95% confidence intervals (CI) were used. We conducted all analyses using IBM SPSS Statistics (IBM Corporation, Armonk, NY).

## RESULTS

There were 57 patients who were initially considered eligible, with 38 who consented, enrolled, and were analyzed for outcomes. Twelve individuals refused participation on CCP days, and seven on ITC days. Baseline characteristics were similar between groups (Table 1). Two patients in each group were lost to follow-up prior to obtaining the 60-minute pain score. Study participants’ length of stay was similar in both groups (ITC = 117 minutes [± 84] vs CCP = 109 minutes [± 56], *P* = 0.97).

**Figure 1:**
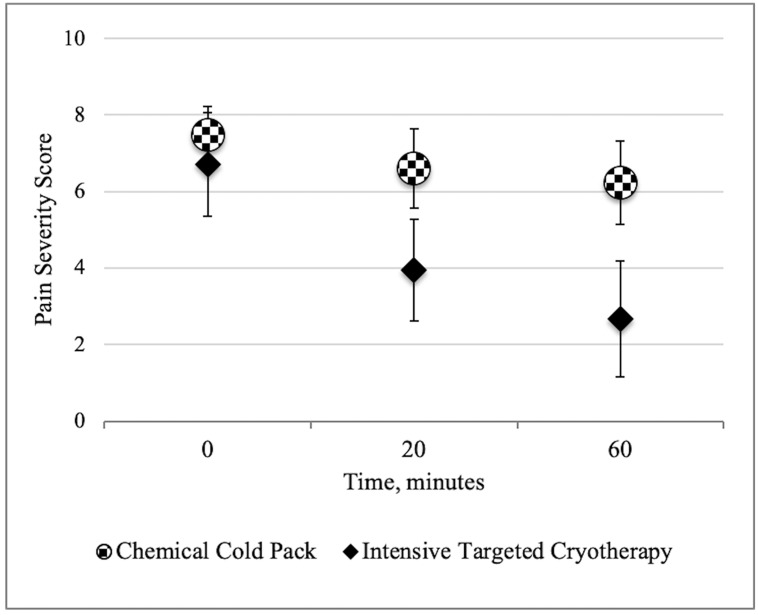
Pain difference between the study arms, with group means 95% confidence interval shown for each group at all time points.

Initial pain scores were similar between the ITC (6.7 [95% CI, 5.4–8.0]) and CCP groups (7.5 [95% CI, 6.8–8.2]) *P* = 0.31). The ITC group achieved statistically significant pain reduction at 20 minutes (2.1 [95% CI, 1.31–2.94] vs 0.9 [95% CI, 0.25–1.51), *P* < 0.05) and 60 minutes (2.7 [95% CI 1.59–3.74] vs 1.2 [CI, 0.62 – 1.69], *P* < 0.05) (Figure 1). At 60 minutes, 11 participants (65%) of the ITC group achieved significant pain reduction compared with 7 (33%) with CCP, with a number needed to treat (NNT) of 3.2 for ITC to provide significant pain relief.

There was no significant difference between the groups with non-opioid analgesic or non-steroidal inflammatory use (ITC = 7 [41%] vs CCP = 10 [48%], *P* = 0.69) nor in absence of pharmacologic analgesia during the visit (ITC = 10 [59%] vs CCP = 7 [33%], *P* = 0.17). The likelihood of a participant receiving opioid prescriptions was not correlated with injury type (*P* = 0.47). One (6%) ITC participant received opioids in the ED compared to seven (33%) in the CCP group (*P* < 0.05), with a NNT of 3.6 for ITC to reduce one patient receiving opioids.

Those who received a discharge prescription for opioids had significantly higher pain scores at 60 minutes (7.3 ± 2.2 vs 4.1 ± 2.7, *P* < 0.05), but prescriptions were not significantly associated with injury type (*P* = 0.47). There was no statistically significant difference in discharge opioid prescriptions (ITC = 4 [24%] vs CCP = 9 [43%], *P* = 0.23) nor when prescriptions included benzodiazepines (ITC = 6 [35%] vs CCP = 11 [52%], *P* = 0.19]). Non-opioid discharge prescriptions were provided to four (24%) participants in the ITC group and five (24%) participants in the CCP group, with similar absence of discharge prescriptions between the groups (ITC = 9 ([53%] vs CCP = 7 [33%], *P* = 0.22]). There were no adverse events in either group and the respective cryotherapy modality was in position at the site of injury at 20 minutes for all participants before being removed by staff.

## DISCUSSION

We found that crushed, wetted ice bags provided greater analgesia for acute musculoskeletal injuries compared to chemical cold packs. This common cryotherapy application is ubiquitous in sports medicine and is easily applicable to ED patients. Furthermore, as pain management is one of the patient experience care domains directly tied to federal hospital reimbursement, optimal cryotherapy is an implementable protocol that could improve both customer satisfaction and hospital remuneration. There have been no reported adverse side effects of cryotherapy in published clinical trials, underscoring the safety of this treatment modality when properly used.^3^

The study participants who received ITC had significantly less opioid utilization than those with CCP. And while there were half the number of opioid prescriptions in the ITC group, the small number of individuals who received opioids overall limited insight into this relationship. Although this study was not powered to evaluate the impact of ICT on opioid prescriptions, with demonstrable analgesia by ITC, this cryotherapy application may have led to decreased patient prescription requests. Prescription opioids have abuse susceptibility similar to heroin,^17^ and have helped fuel one of the nation’s most pressing public health challenges. As short-course opioid therapy is associated with recurrent opioid use and may contribute to development of addiction,^18^ it is reasonable that improved analgesia through optimal cryotherapy could help mitigate potential opioid abuse. Larger studies are needed to further elucidate the effect of optimal cryotherapy on opioid prescriptions.

## LIMITATIONS

Although the trial met its pre-specified enrollment threshold, the primary limitation was its relatively small sample size and single-center design that limited subgroup analyses. By design, we did not focus the cryotherapy on specific anatomic locations or presumed diagnoses that may have responded better to one cryotherapy treatment over another. Similarly, the heterogenous injury pattern limited our ability to draw practice-changing conclusions regarding the use of cryotherapy for specific injuries. While individual provider practice may have confounded the outcomes, with three months of data collection and a large number of treating physicians, this is unlikely. Describing early analgesic effect within the CCP group may have been missed by not measuring pain scores at shorter intervals (eg, at 10 or 15 minutes). However, our primary outcome of pain at 60 minutes was selected to allow for tissue temperature normalization and greater clinical relevance.

It was not logistically feasible to include a placebo arm, but as participants were blinded to which treatment arm was experimental and which was an active control, this unlikely affected the primary outcome. Possible selection bias was mitigated by allocating participants to treatment arms based on the day of the month, and by separating the roles of various members of the research team. Specifically, the research assistants were trained to enroll and consent participants for the study, so long as the inclusion criteria were met (and the exclusion criteria were not), regardless of the day of the month. These research assistants were blinded to the hypotheses of the study and were undergraduate students with training in Health Insurance Portability and Accountability Act requirements and informed consent (but without significant medical training). Although the chart reviewers were blinded to treatment received, it was impossible to blind the treating physician; thus, the potential impact this had on analgesics is unknown. However, as no treating physicians were aware that analgesia was a studied variable in this trial, it was unlikely that awareness of cryotherapy type effected opioid usage.

Although participants were enrolled in triage prior to a physician encounter, the time between cryotherapy application and physician interaction was not standardized and could have influenced pain severity and the likelihood of opioid administration. Finally, because the CCP did not likely stay as cold as the crushed ice for the full 20 minutes of application, the beneficial effect shown could be explained by the duration of effective cryotherapy received by participants. Measuring pain scores at 60 minutes attempted to account for tissue temperature equilibration, but our methodology may underestimate the analgesic potential of CCPs.

## CONCLUSION

Intensive targeted cryotherapy provided more effective analgesia than chemical cold packs for patients with acute musculoskeletal injuries in the ED and may contribute to lower opioid usage.

## Figures and Tables

**Table 1:** Participant characteristics.

Participant Characteristics	Chemical Cold Packs N, (%)		Intensive Targeted Cryotherapy N, (%)		*P-value*
Demographics					
Number	21	(55)	17	(45)	0.57
Women	12	(57)	13	(76)	0.21
Age, (years) Mean (± SD)	33	(± 12)	35	(± 14)	0.61
Cryotherapy duration, (minutes) Mean (± SD)	20	(± 1.6)	21	(± 2.9)	0.20
Clinical Characteristics					
Injury Site					
Ankle	3	(14)	3	(18)	0.78
Arm	1	(5)	0	(0)	0.36
Back	1	(5)	2	(12)	0.43
Clavicle	1	(5)	0	(0)	0.36
Coccyx	1	(5)	0	(0)	0.36
Elbow	2	(10)	0	(0)	0.19
Foot	1	(5)	2	(12)	0.43
Groin	0	(0)	1	(6)	0.26
Hand	1	(5)	1	(6)	0.88
Knee	3	(14)	4	(29)	0.46
Neck	3	(14)	0	(0)	0.10
Shoulder	1	(5)	2	(12)	0.43
Toe	0	(0)	1	(6)	0.26
Rib	1	(5)	0	(0)	0.36
Wrist	2	(10)	0	(0)	0.19
Diagnosis					
Fracture	4	(19)	4	(24)	0.74
Sprain or strain	2	(10)	5	(29)	0.12
Torn ligament	3	(14)	1	(6)	0.40
Contusion or pain	12	(57)	7	(41)	0.33

*N*, number; *SD*, standard deviation.
